# Development and validation of a nomogram for predicting intrapartum fever in parturients with epidural analgesia

**DOI:** 10.3389/fmed.2025.1668597

**Published:** 2025-10-06

**Authors:** Dong-ge Niu, Lei Wang, Rui-yuan Wei, Ju Li, Yue-ning Zhu, Hua Zhang, Lan Yao

**Affiliations:** ^1^Department of Anesthesiology, Peking University International Hospital, Beijing, China; ^2^Department of Obstetrics and Gynecology, Peking University International Hospital, Beijing, China; ^3^Research Center of Clinical Epidemiology, Peking University Third Hospital, Beijing, China

**Keywords:** intrapartum fever, epidural analgesia, inflammation, SII, nomogram

## Abstract

**Background:**

Intrapartum fever is a common complication during labor that may adversely affect both maternal and neonatal outcomes. However, its underlying multifactorial etiology remains incompletely understood.

**Objective:**

This study aimed to investigate the clinical and inflammatory factors associated with intrapartum fever and to construct a predictive model for individualized risk assessment.

**Methods:**

In this retrospective cohort study, we analyzed clinical data from 1,692 parturients who received epidural analgesia during term singleton vaginal delivery between September 2019 and October 2021. Participants were randomly divided into training and validation sets (7:3). Independent predictors of intrapartum fever were identified using multivariate logistic regression. A nomogram was constructed and evaluated through receiver operating characteristic (ROC) curves, calibration plots, and decision curve analysis (DCA).

**Results:**

Intrapartum fever occurred in 5.0% of cases. Seven independent predictors were identified, including admission body temperature, premature rupture of membranes (PROM), duration of the first stage of labor, number of vaginal examinations, interleukin-6 (IL-6), systemic immune-inflammation index (SII), and neutrophil-to-lymphocyte ratio (NLR). Among these, IL-6 and SII demonstrated the strongest predictive performance, with SII showing a higher AUC than NLR (0.846 vs. 0.716). The final nomogram incorporating six variables achieved excellent discrimination (AUC = 0.910 in the training set and 0.906 in the validation set) and demonstrated good calibration and clinical utility.

**Conclusion:**

Intrapartum fever is associated with both obstetric stress and systemic inflammation. The proposed nomogram, integrating readily available clinical and inflammatory markers, enables individualized risk assessment and may assist in early identification of high-risk parturients, supporting timely clinical intervention during labor.

## Introduction

Childbirth is widely regarded as one of the most painful experiences in a woman’s life. Therefore, effective pain relief is a critical component of obstetric care (1). Epidural labor analgesia is considered the gold standard for intrapartum pain management, as it is associated with higher maternal satisfaction and favorable maternal and neonatal safety profiles. However, its widespread use has also been linked to several adverse outcomes, such as prolonged first and second stages of labor, instrumental delivery, urinary retention, perineal laceration, and intrapartum fever (2, 3).

Intrapartum fever has been associated with a variety of obstetric risk factors, including nulliparity, labor induction, prolonged labor duration, group B streptococcus colonization, and the use of epidural analgesia (4). Fusi et al. were among the first to report that women receiving epidural analgesia during labor had a higher incidence of intrapartum fever (5). Since then, numerous randomized controlled trials and retrospective cohort studies have confirmed this association. However, the underlying mechanisms remain controversial. Some studies suggest that maternal fever is primarily related to the duration of labor and the cumulative exposure to epidural analgesic agents (6, 7), while others have found increased rates of fever in epidural users even after adjusting for labor duration (8, 9).

Given the multifactorial etiology of intrapartum fever, a comprehensive assessment of both clinical and inflammatory factors is essential. Therefore, the present study aimed not only to identify independent obstetric and inflammatory predictors of intrapartum fever in parturients receiving epidural analgesia, but also to compare the predictive performance of commonly used inflammatory biomarkers such as IL-6, systemic immune-inflammation index (SII), and neutrophil-to-lymphocyte ratio (NLR). Furthermore, we sought to integrate these predictors into a user-friendly nomogram and to evaluate its discrimination, calibration, and clinical utility in both training and validation cohorts. By doing so, this study intended to provide clinicians with a reliable tool for individualized risk stratification, early recognition of high-risk women, and timely intrapartum management.

## Methods

### Ethics statement

Since this study was a retrospective analysis using anonymized data, individual informed consent was waived. The protocol was approved by the Ethics Committee of the Peking University International Hospital (Approval No. 2024-KY-0003-01), and exempted from informed consent requirements under protocol version 2024-KY-0003_1.0.

### Participants

**Figure 1:**
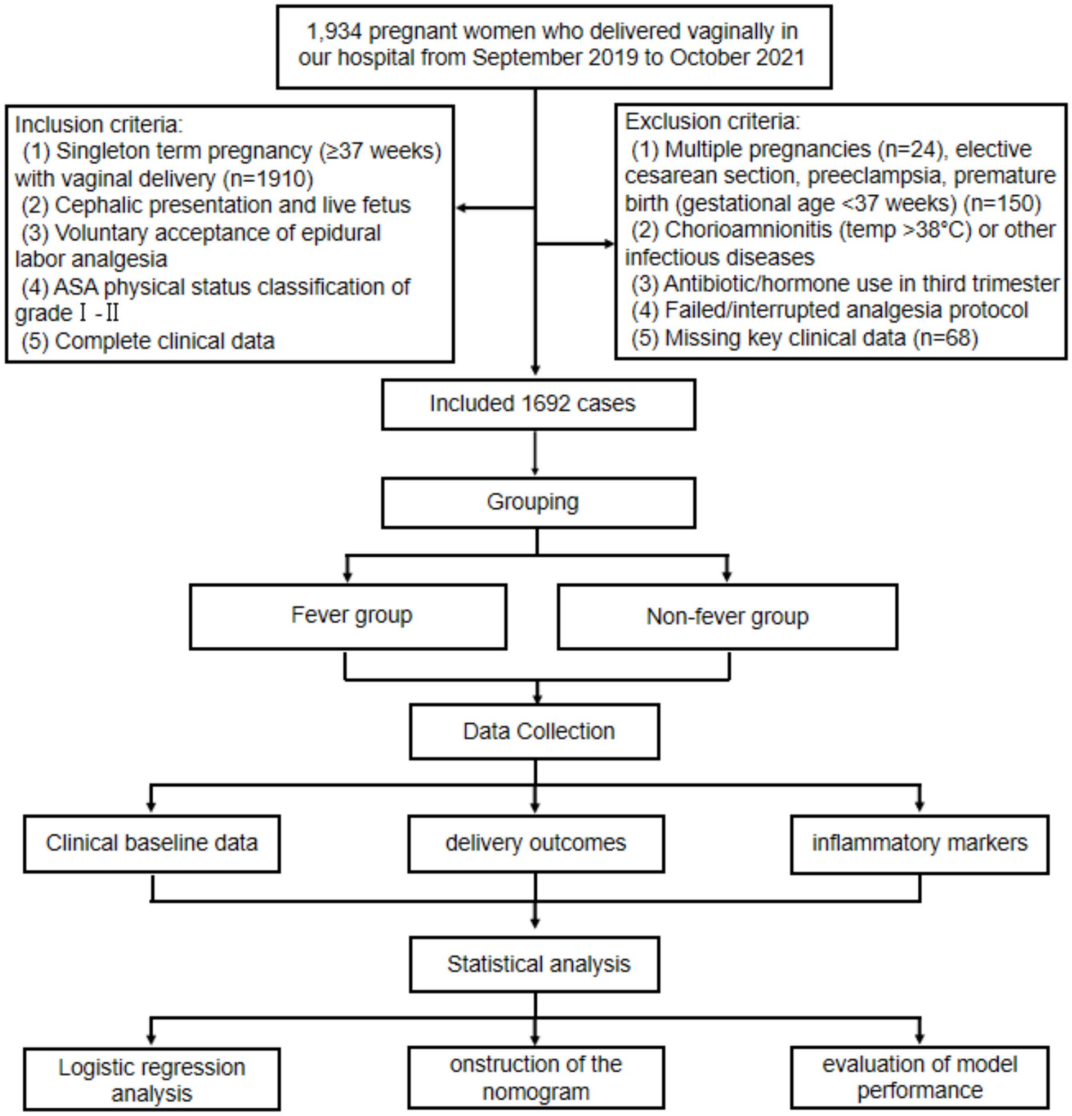
Flowchart of participant selection, grouping, and study procedures.

This retrospective study included parturients who underwent vaginal delivery at Peking University International Hospital between September 2019 and October 2021. A total of 1934 pregnant women were initially screened. Inclusion criteria were as follows: (1) singleton term pregnancy (≥37 weeks) with vaginal delivery; (2) cephalic presentation and live fetus; (3) voluntary epidural labor analgesia; (4) ASA physical status grade I–II; and (5) complete clinical records. Exclusion criteria included: (1) multiple pregnancies; (2) cesarean delivery, preeclampsia, or preterm birth; (3) chorioamnionitis or other infectious diseases; (4) antibiotic or hormone use in the third trimester; (5) failed or interrupted analgesia; and (6) missing key clinical data. Among above, Chorioamnionitis was excluded based on comprehensive clinical assessment, including maternal intrapartum fever accompanied by maternal or fetal tachycardia, uterine tenderness, or foul-smelling amniotic fluid, in line with established obstetric diagnostic criteria (10). Histopathological confirmation was not routinely available for all placentas in this retrospective cohort; therefore, cases with documented clinical suspicion or diagnosis of chorioamnionitis in the medical record were excluded from analysis. After applying these criteria, 1,692 eligible participants were included. To enable model construction and internal validation, participants were randomly assigned to training and validation sets in a 7:3 ratio. A detailed selection flowchart is presented in Figure 1. This study was approved by the Ethics Committee of the Peking University International Hospital of Beijing (2024-KY-0003-01), and was exempted on the basis of an anonymous analysis (Exemption of informed consent number: 2024-KY-0003_1.0).

### Intervention

Epidural analgesia was administered upon maternal request when cervical dilation reached approximately 1–2 cm. The analgesic protocol included a 10 mL bolus of 0.1% ropivacaine combined with 5 mcg fentanyl, followed by continuous infusion of 0.1% ropivacaine with 0.5 mcg/mL fentanyl at 10 mL/h. Additional 10 mL boluses were allowed with a 20-min lockout interval.

### Data collection

Clinical data were retrospectively extracted from electronic medical records and included information on labor progression, maternal characteristics, and neonatal outcomes. Intrapartum fever was defined as an axillary temperature ≥38 °C, confirmed by two measurements taken 30 min apart (10). The following variables were collected at admission or during labor: maternal age, body mass index (BMI), gestational age, parity, admission temperature, adverse pregnancy history, hypertensive disorders, diabetes, thyroid dysfunction (hypo- or hyperthyroidism), HELLP syndrome, anemia, premature rupture of membranes (PROM), artificial rupture of membranes, GBS colonization status, duration of epidural analgesia, oxytocin use, durations of the first and second stages of labor, number of vaginal examinations, and grade III amniotic fluid contamination. Neonatal outcomes included birth weight, sex, 1-min Apgar score ≤7, neonatal jaundice index, and NICU admission. Inflammatory markers were obtained from admission blood samples and included White blood cells (WBC) count, neutrophil count, lymphocyte count, hemoglobin, platelet count, C-reactive protein (CRP), interleukin-6 (IL-6), tumor necrosis factor-*α* (TNF-α), systemic immune-inflammation index (SII), neutrophil-to-lymphocyte ratio (NLR), and platelet-to-lymphocyte ratio (PLR). These indices were calculated as follows: SII = (neutrophil count × platelet count)/lymphocyte count; NLR = neutrophil count/lymphocyte count; PLR = platelet count/lymphocyte count.

### Statistical analysis

Statistical analyses were performed using SPSS version 4.0.3 (SPSS Inc., Chicago, IL., United States). The Shapiro–Wilk test was used to assess normality of continuous variables. Normally distributed data were expressed as mean ± standard deviation (SD) and compared using the independent-sample *t*-test. Non-normally distributed data were presented as median (interquartile range) and compared using the Wilcoxon rank-sum test. Categorical variables were described as frequency (percentage) and compared using the chi-square (χ^2^) test or Fisher’s exact test. To identify predictors of intrapartum fever, univariate and multivariate logistic regression analyses were performed. Continuous predictors (such as age, BMI, admission temperature, labor durations, and inflammatory markers) were entered into the models as continuous variables rather than being arbitrarily categorized, in order to preserve statistical power and avoid information loss. When appropriate, least absolute shrinkage and selection operator (LASSO) regression was used for variable selection. A nomogram was constructed based on the final predictors. Model performance was evaluated in both training and validation sets using ROC curves (area under the curve, AUC), calibration curves, and decision curve analysis (DCA). A two-tailed *p* < 0.05 were considered statistically significant.

## Results

### Clinical characteristics of the study population

A total of 1,692 parturients who received epidural analgesia during term singleton vaginal delivery were included, with 1,110 allocated to the training set and 582 to the validation set (Table 1). The overall median maternal age was 29 years, BMI was 26.29 kg/m^2^, and gestational age was 39 weeks. Common comorbidities included hypertension (47.9%), diabetes (45.1%), hypothyroidism (44.6%), and hyperthyroidism (47.9%). Labor-related variables such as duration of analgesia (median 8 h), oxytocin use (47.2%), and labor stage durations were generally comparable between groups. However, the validation set showed a higher incidence of grade III amniotic fluid contamination (14.8% vs. 3.2%), 1-min Apgar ≤7 (15.1% vs. 8.7%) and NICU admissions (10.7% vs. 4.4%). Inflammatory markers, including WBC, IL-6, TNF-*α*, SII, NLR, and PLR, were evenly distributed across both sets. No significant baseline differences were observed for other maternal, obstetric, or neonatal variables, indicating effective randomization.

**Table 1:** Baseline clinical characteristics of participants in training and validation sets.

	Overall (*N* = 1,692)	Training set (*N* = 1,110)	Validation set (*N* = 582)
Baseline data			
Age	29 (27–30)	29 (27–30)	28.5 (26–30)
BMI	26.29 (24.39–28.31)	26.35 (24.39–28.32)	26.16 (24.39–28.26)
Gestational age	39 (38–40)	39 (38–40)	39 (38–40)
Number of deliveries	1 (0–1)	0 (0–1)	1 (0–1)
Admission temperature	36.3 (35.6–37)	36.2 (35.6–36.9)	36.5 (35.7–37.1)
Adverse pregnancy history	172 (10.2)	116 (10.5)	56 (9.6)
Hypertension	811 (47.9)	518 (46.7)	293 (50.3)
Diabetes	763 (45.1)	466 (42.0)	297 (51.0)
Hypothyroidism	754 (44.6)	474 (42.7)	280 (48.1)
Hyperthyroidism	810 (47.9)	521 (46.9)	289 (49.7)
HELLP syndrome	750 (44.3)	474 (42.7)	276 (47.6)
Anemia	756 (44.7)	492 (44.3)	264 (45.6)
Premature rupture of membranes (PROM)	807 (47.7)	512 (46.1)	295 (50.7)
Rupture of artificial membranes	817 (48.3)	532 (47.9)	285 (49.0)
GBS (positive/negative)	791/901	491/619	300/282
Duration of analgesia	8 (7–9)	8 (7–9)	8 (7–9.25)
Oxytocin use	799 (47.2)	509 (45.9)	290 (49.8)
Duration of first stage of labor	390 (240–590)	345 (240–530)	480 (287.5–660)
Duration of second stage of labor	45 (23–82)	38 (21–70)	63 (30–100)
Number of vaginal examinations	3 (2–4)	3 (2–4)	3 (2–4)
Amniotic fluid contamination grade III	121 (7.2)	35 (3.2)	86 (14.8)
Delivery outcome			
Birth weight	3,320 (3090–3,580)	3,320 (3090–3,570)	3,325 (3080–3,600)
Gender	864/828	575/535	289/293
1-min Apgar score ≤7	185	97 (8.7)	88 (15.1)
Neonatal jaundice index	5.43 (4.53–6.43)	6.36 (4.26–6.53)	4.25 (4.02–6.23)
Admission to neonatal ward	111 (6.6)	49 (4.4)	62 (10.7)
Inflammatory markers			
White blood cells (WBC)	7.11 (5.45–8.78)	7.01 (5.4–8.61)	7.27 (5.55–9.12)
Neutrophils	51 (46–56)	51 (46–56)	52 (46–56)
Lymphocytes	46 (42–49)	46 (42–50)	45 (41–49)
Hemoglobin	135 (130–140)	135 (130–140)	135 (129–140)
Platelets	209.54 (192.54–227.15)	210.57 (192.18–226.78)	208.51 (192.59–227.75)
CRP	8.78 (6.73–10.87)	8.87 (6.82–10.91)	8.72 (6.6–10.76)
IL-6	4.16 (2.7–5.65)	4.05 (2.61–5.51)	4.34 (2.8–5.94)
TNF-α	7.7 (4.88–10.35)	7.52 (4.77–10.19)	8.07 (5.25–10.66)
SII	231.49 (204.73–364.47)	229.02 (203.24–260.68)	235.13 (207.18–270.41)
NLR	1.11 (1–1.83)	1.1 (0.98–1.22)	1.12 (1–1.28)
PLR	4.61 (4.13–5.14)	4.59 (4.12–5.06)	4.66 (4.17–5.31)

### Comparison between fever and non-fever groups in the training set

Among the 1,110 parturients in the training set, 56 (5.0%) developed intrapartum fever. Compared to the non-fever group (*N* = 1,054), the fever group exhibited significant differences in multiple maternal, obstetric, and inflammatory parameters (Table 2). In terms of baseline characteristics, the fever group had a significantly higher admission temperatures (median 38.6 °C vs. 36.2 °C, *p* < 0.001), a higher rate of adverse pregnancy history (37.5% vs. 9.0%, *p* < 0.001), and a greater prevalence of anemia (60.7% vs. 43.5%, *p* = 0.011). No significant differences were observed in age, BMI, gestational age, or comorbidities such as hypertension, diabetes, or thyroid disorders. Regarding obstetric characteristics, the fever group experienced a significantly longer duration of labor analgesia (median 9 vs. 8 h, *p* < 0.001), higher oxytocin use (69.6% vs. 44.6%, *p* < 0.001), prolonged first-stage labor (530 vs. 330 min, *p* < 0.001) and second-stage labor (71.5 vs. 38 min, *p* < 0.001), as well as a higher number of vaginal examinations (median 3 vs. 1, *p* < 0.001). The PROM was also significantly higher in the fever group (82.1% vs. 44.2%, *p* < 0.001).

**Table 2:** Comparison of variables between the fever group and the non-fever group in the training set.

	No fever (*N* = 1,054)	Fever (*N* = 56)	Statistics	*P*
Baseline data				
Age	29 (27–30)	28 (26–30)	−1.652	0.099
BMI	26.35 (24.28–28.32)	27.16 (25.14–29.22)	−1.766	0.077
Gestational age	39 (38–40)	39 (38.25–40.00)	−0.942	0.346
Number of deliveries	0 (0–1)	1 (0–1)	−1.123	0.261
Admission temperature	36.2 (35.6–36.8)	38.6 (38–39.2)	−12.634	<0.001
Adverse pregnancy history	95 (9.0)	21 (37.5)	46.11	<0.001
Hypertension	492 (46.7)	26 (46.4)	0.001	0.971
Diabetes	440 (41.7)	26 (46.4)	0.479	0.489
Hypothyroidism	445 (42.2)	29 (51.8)	1.989	0.158
Hyperthyroidism	498 (47.2)	23 (41.1)	0.815	0.367
HELLP syndrome	447 (42.4)	27 (48.2)	0.732	0.392
Anemia	458 (43.5)	34 (60.7)	6.42	0.011
Premature rupture of membranes (PROM)	466 (44.2)	46 (82.1)	30.786	<0.001
Rupture of artificial membranes	505 (47.9)	27 (48.2)	0.002	0.965
GBS (positive/negative)	470/584	21/35	1.084	0.298
Duration of analgesia	8 (7–9)	9 (7–10)	−7.883	<0.001
Oxytocin use	470 (44.6)	39 (69.6)	13.44	<0.001
Duration of first stage of labor	330 (240–510)	530 (343.5–650)	−4.518	<0.001
Duration of second stage of labor	38 (21–67)	71.5 (45–127)	−4.568	<0.001
Number of vaginal examinations	1 (1–2)	3 (3–4)	−11.468	<0.001
Grade III amniotic fluid contamination	32 (3.0)	3 (5.4)	0.938	0.333
Delivery outcome				
Birth weight	3,500 (3155–3697.5)	3,320 (3070–3,570)	1.263	0.102
Sex (male/female)	507/547	28/28	0.077	0.782
1-min Apgar score ≤7	56 (5.3)	41 (73.2)	307.415	<0.001
Neonatal jaundice index	6.53 (4.26–8.96)	5.53 (3.26–9.52)	1.236	0.126
Number of cases admitted to the neonatal ward	20 (1.9)	29 (51.8)	313.643	<0.001
Inflammatory indicators				
White blood cells (WBC)	6.86 (5.33–8.35)	11.76 (10.4–13.46)	−12.213	<0.001
Neutrophils	51 (46–55)	75.5 (72–80.25)	−12.645	<0.001
Lymphocytes	46 (43–50)	32 (29–36.25)	−12.637	<0.001
Hemoglobin	135 (130–140)	134 (131–139)	−0.222	0.824
Platelets	211.52 (193.25–227.3)	204.83 (189.28–224.59)	−1.3	0.194
CRP	8.71 (6.67–11.94)	8.91 (7.35–10.48)	−0.502	0.616
IL-6	3.89 (2.57–9.3)	8.95 (7.47–11.68)	−12.416	<0.001
TNF-α	7.17 (4.59–14.56)	15.32 (11.37–18.19)	−10.991	<0.001
SII	226.62 (202.22–455.99)	468.36 (426.86–531.11)	−12.625	<0.001
NLR	1.08 (0.98–2.89)	2.27 (2.13–2.63)	−12.626	<0.001
PLR	4.54 (3.11–6.97)	6.23 (5.76–6.87)	−11.7	<0.001

In terms of neonatal outcomes, the fever group had a markedly higher incidence of 1-min Apgar scores ≤7 (73.2% vs. 5.3%, *p* < 0.001) and NICU admissions (51.8% vs. 1.9%, *p* < 0.001), suggesting a strong association between maternal fever and adverse neonatal status. For inflammatory markers, the fever group exhibited significantly elevated levels of WBC (11.76 vs. 6.86 × 10^9^/L), neutrophil ratio (75.5% vs. 51%), IL-6 (8.95 vs. 3.89 pg./mL), TNF-*α* (15.32 vs. 7.17 pg./mL), SII (468.36 vs. 226.62), NLR (2.27 vs. 1.08), and PLR (6.23 vs. 4.54), all with *p* < 0.001. Conversely, lymphocyte counts were significantly lower in the fever group (32 vs. 46, *p* < 0.001). These results suggest a robust systemic inflammatory response in patients with intrapartum fever.

### Logistic regression analysis of risk factors for intrapartum fever

Univariate logistic regression analysis identified multiple factors significantly associated with intrapartum fever (Table 3), including elevated admission temperature, anemia, PROM, prolonged labor analgesia, oxytocin use, longer first stage labor, more frequent vaginal examinations, and elevated inflammatory markers such as WBC, neutrophils, IL-6, TNF-*α*, SII, NLR, and PLR (all *p* < 0.05). In multivariate analysis, seven variables remained independently associated with intrapartum fever: admission temperature (OR = 1.569, 95% CI: 1.023–3.126, *p* = 0.013), PROM (OR = 1.596, 95% CI: 1.126–3.263, *p* = 0.031), duration of the first stage of labor (OR = 3.122, 95% CI: 2.163–6.523, *p* < 0.001), number of vaginal examinations (OR = 1.596, 95% CI: 1.023–1.999, *p* = 0.031), IL-6 (OR = 3.201, 95% CI: 2.162–4.596, *p* < 0.001), SII (OR = 3.522, 95% CI: 2.456–6.955, *p* < 0.001), and NLR (OR = 1.988, 95% CI: 1.456–6.596, *p* < 0.001). Other factors such as anemia, oxytocin use, WBC count, TNF-*α*, and PLR were no longer significant after adjustment.

**Table 3:** Logistic regression analysis table.

	Single factor		Multifactorial		
	95%CI	*P*	β coefficient	95%CI	*P*
Baseline data					
Admission temperature	2.312 (1.126–4.152)	0.001	0.45	1.569 (1.023–3.126)	0.013
Anemia	1.526 (1.023–2.365)	0.016	0.02	1.023 (0.899–1.562)	0.129
Premature rupture of membranes	2.152 (1.256–3.562)	0.029	0.47	1.596 (1.126–3.263)	0.031
Duration of analgesia	2.362 (1.269–5.621)	0.035	0.02	1.023 (0.986–1.056)	0.321
Oxytocin use	1.263 (1.023–1.956)	0.042	0.01	1.011 (0.999–1.269)	0.452
Duration of first stage of labor	4.263 (1.596–6.596)	<0.001	1.14	3.122 (2.163–6.523)	<0.001
Number of vaginal examinations	2.362 (1.569–5.623)	0.016	0.47	1.596 (1.023–1.999)	0.031
Inflammatory markers					
White blood cells (WBC)	3.126 (1.999–6.899)	0.026	0.02	1.023 (0.975–1.999)	0.269
Neutrophils	4.523 (1.685–15.632)	0.036	0.94	2.563 (0.759–8.599)	0.456
IL-6	3.263 (2.456–8.955)	<0.001	1.16	3.201 (2.162–4.596)	<0.001
TNF-α	1.236 (1.001–2.123)	<0.001	0.26	1.296 (0.756–6.235)	0.523
SII	4.523 (2.412–8.999)	<0.001	1.26	3.522 (2.456–6.955)	<0.001
NLR	2.659 (1.867–8.974)	<0.001	0.69	1.988 (1.456–6.596)	<0.001
PLR	1.255 (1.002–1.695)	0.04	0.02	1.022 (0.977–3.563)	0.326

These findings highlight the independent contributions of both obstetric factors (PROM, prolonged labor, vaginal examinations) and systemic inflammation—particularly IL-6, SII, and NLR—to the development of intrapartum fever under epidural analgesia.

### Diagnostic performance of SII and NLR for predicting intrapartum fever

**Figure 2:**
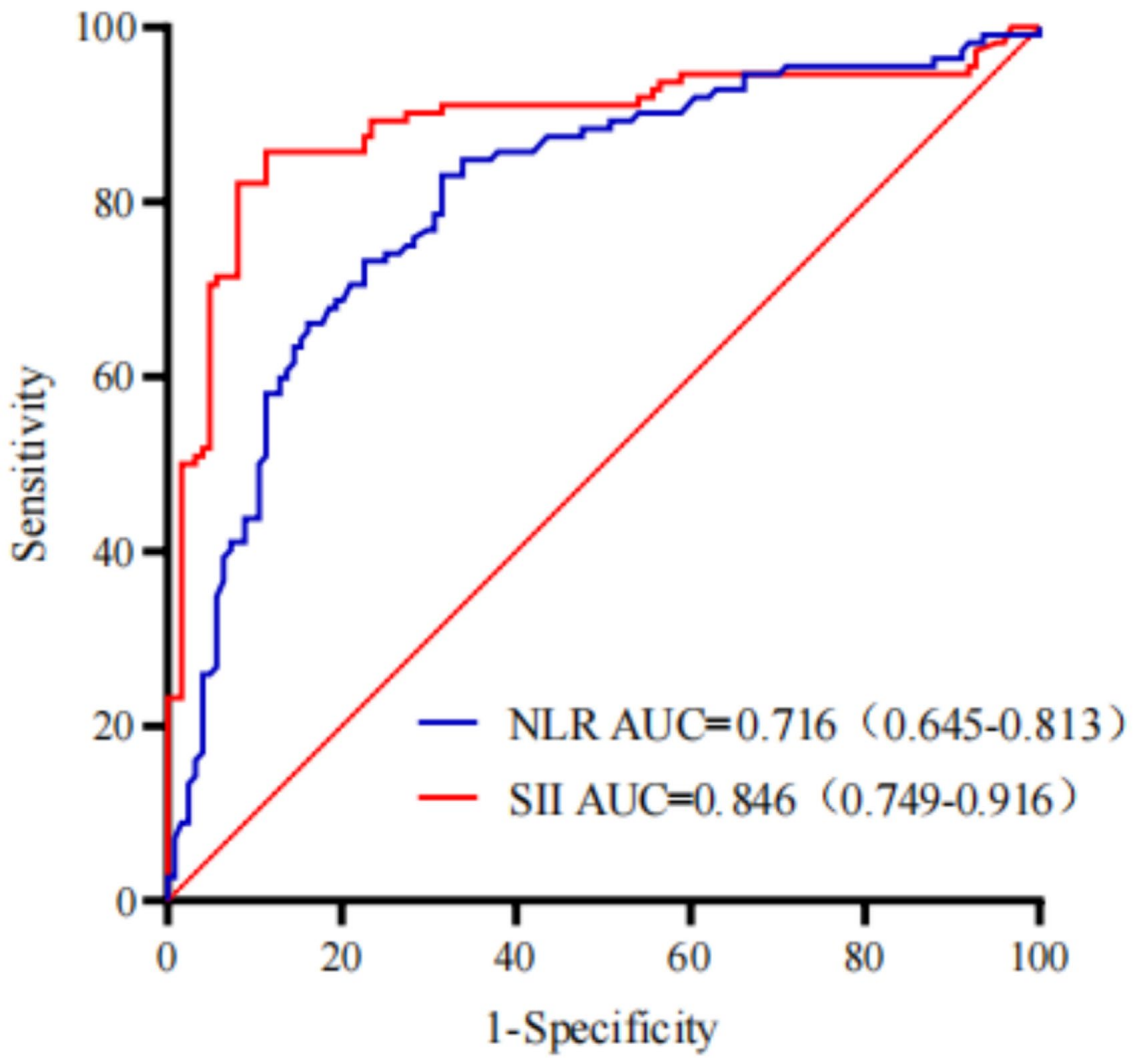
The ROC curves comparing the diagnostic performance of the SII and NLR for predicting intrapartum fever.

The ROC analysis was performed to compare the predictive ability of SII and NLR for intrapartum fever. As shown in Figure 2, the AUC for SII was 0.846 (95% CI: 0.749–0.916), whereas that for NLR was 0.716 (95% CI: 0.645–0.813). The ROC curve of SII consistently outperformed that of NLR across all thresholds, indicating that SII had superior discriminative ability as a single inflammatory predictor.

### Nomogram for individualized prediction of intrapartum fever

**Figure 3:**
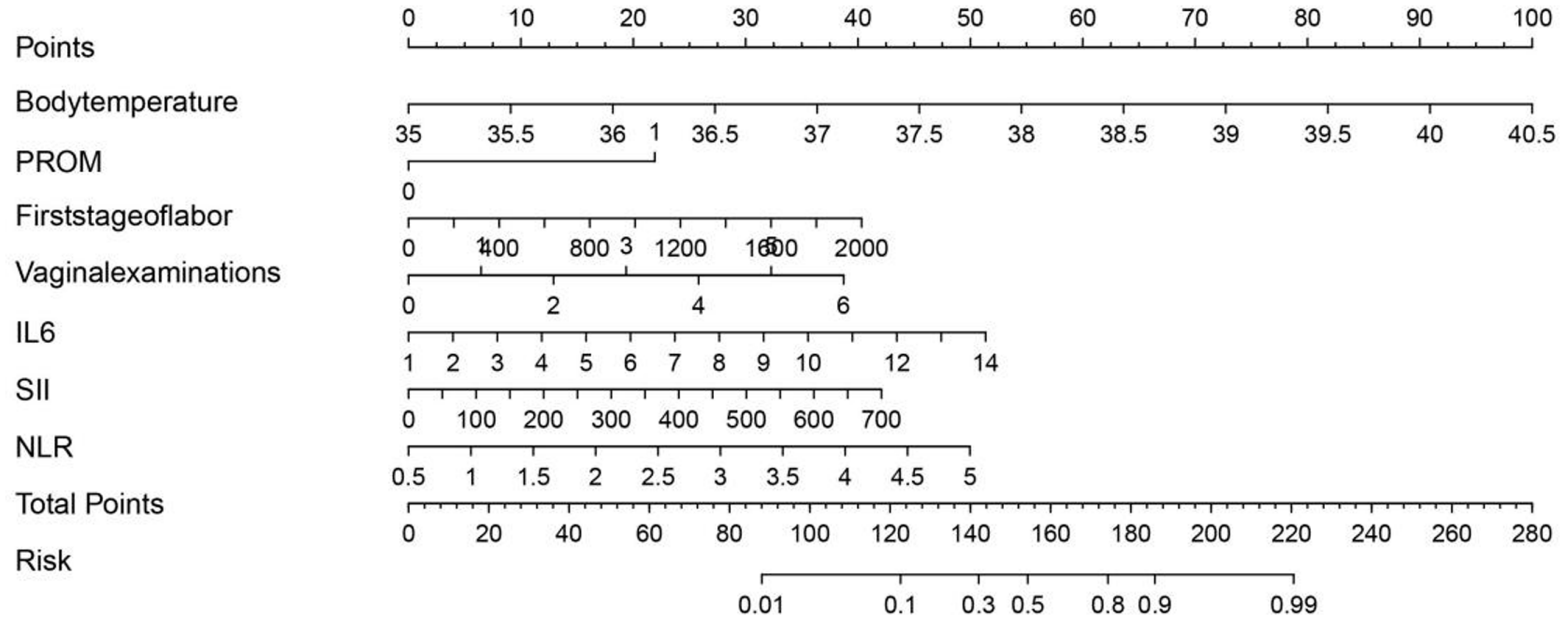
Nomogram for predicting intrapartum fever in parturients receiving epidural analgesia, based on body temperature, PROM, first-stage labor duration, number of vaginal examinations, IL-6, SII, and NLR.

Based on the final multivariate logistic regression model, a nomogram was developed incorporating seven independent predictors: body temperature, PROM, first-stage labor duration, number of vaginal examinations, IL-6, SII, and NLR (Figure 3). The total score ranges from 0 to approximately 280 and corresponds to a predicted risk of intrapartum fever ranging from 0.01 to 0.99. Among all predictors, IL-6 and SII contributed the most to the overall score, followed by body temperature and NLR, indicating their relatively greater importance in risk estimation. PROM, labor duration, and vaginal examination frequency had moderate contributions. This tool enables quantitative risk prediction for individual parturients by summing the points assigned to each variable and mapping the total score to the corresponding risk probability.

### Performance evaluation of the predictive model

**Figure 4:**
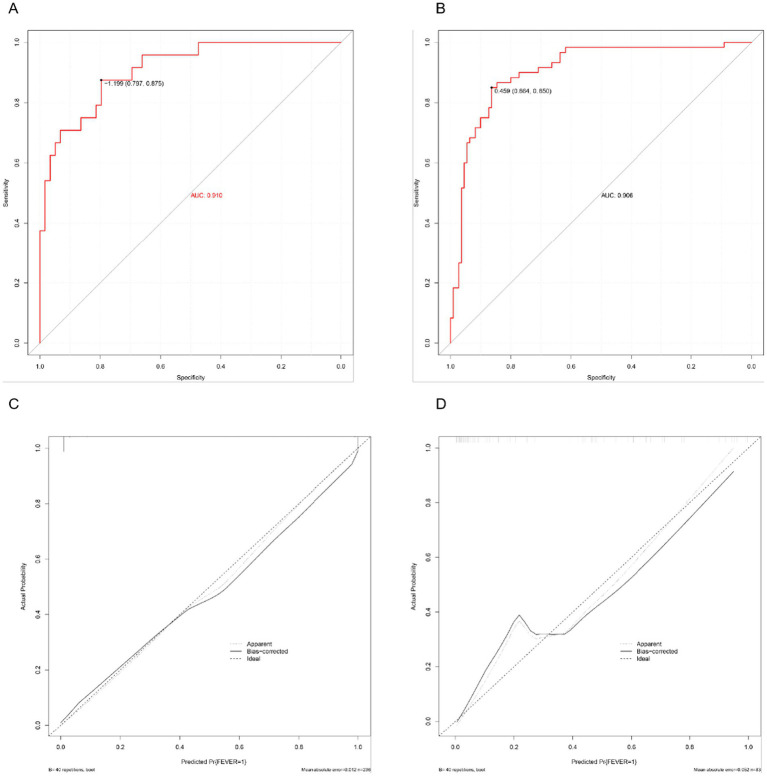
Performance evaluation of the predictive nomogram. **(A)** ROC curve in the training set. **(B)** ROC curve in the validation set. **(C)** Calibration curve in the training set. **(D)** Calibration curve in the validation set.

Discrimination performance was assessed using ROC curves. In the training set, the nomogram demonstrated excellent discriminatory ability with an AUC of 0.910 (Figure 4A). In the validation set, the AUC was 0.906 (Figure 4B), indicating strong generalizability and minimal risk of overfitting. Calibration was evaluated using 1,000 bootstrap resamples. In the training set (Figure 4C), the predicted probabilities aligned closely with observed outcomes across the full range of risk, with the bias-corrected calibration curve nearly overlapping the ideal diagonal. In the validation set (Figure 4D), the calibration plot also showed good agreement, although slight underestimation was observed in higher predicted risk ranges. The mean absolute error (MAE) was 0.012 in the training set and 0.052 in the validation set, supporting the model’s accuracy.

### Decision curve analysis

**Figure 5:**
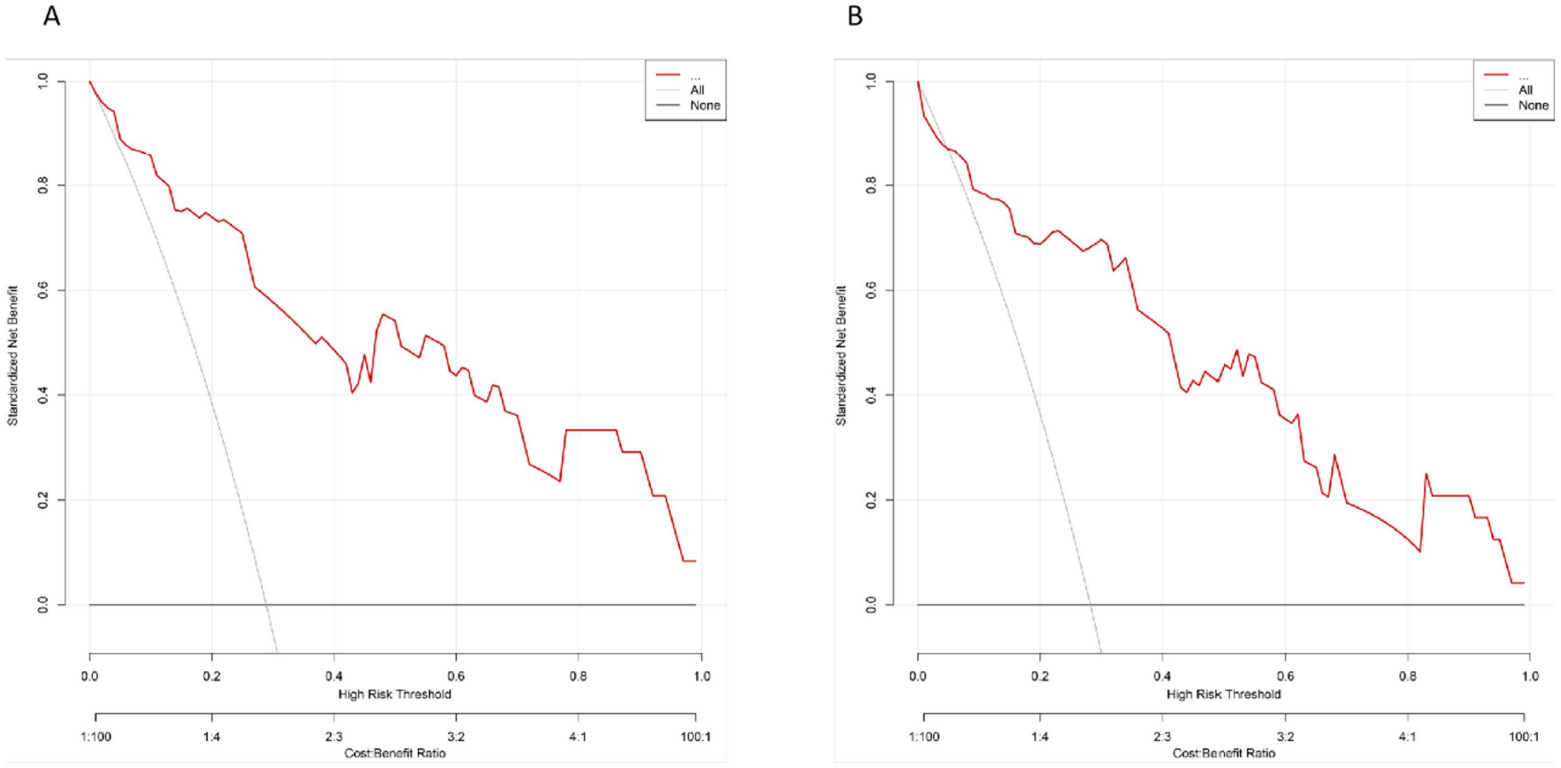
The DCA for the prediction model. **(A)** DCA curve in the training set. **(B)** DCA curve in the validation set. The red curve represents the net benefit of the model across a series of threshold probabilities, compared to the default strategies of treating all patients (gray line) or none (black line).

The DCA was conducted to assess the clinical utility of the nomogram by quantifying the net benefit across various threshold probabilities. As shown in Figure 5A, in the training set, the model consistently outperformed both the “treat-all” and “treat-none” strategies across a clinically relevant threshold range of 0.05–0.80. Similarly, in the validation set (Figure 5B), the model maintained a favorable net benefit between thresholds of 0.05 and 0.75, indicating stable external performance.

These results suggest that within this threshold range, use of the model as a decision-support tool provides greater net clinical benefit compared with indiscriminate or no intervention strategies.

## Discussion

In this retrospective cohort study of 1,692 parturients receiving epidural analgesia, we identified multiple clinical and inflammatory factors independently associated with intrapartum fever. Specifically, elevated admission temperature, PROM, prolonged first-stage labor, increased frequency of vaginal examinations, and elevated inflammatory markers including IL-6, SII, and NLR were significant predictors. Based on these findings, we developed and validated a predictive nomogram that demonstrated excellent discrimination and calibration performance, offering a practical tool for early identification of high-risk individuals.

In addition to the obstetric and inflammatory factors identified in this study, several other determinants of intrapartum fever have been reported in the literature. Prolonged use of epidural analgesia itself has been consistently associated with maternal pyrexia, potentially through non-infectious inflammatory pathways (5, 11). Nulliparity and labor induction have also been recognized as independent risk factors, likely reflecting longer labor duration and increased obstetric interventions (12). Furthermore, maternal colonization with group B streptococcus and placental inflammation have been implicated in the pathogenesis of intrapartum fever, underscoring the multifactorial nature of this condition (7, 10). Collectively, these findings highlight that both infectious and non-infectious processes may contribute to maternal hyperthermia during labor, and should be considered when interpreting our results.

The multifactorial nature of intrapartum fever has been widely recognized, yet few studies have systematically integrated both clinical and inflammatory dimensions in its prediction (11, 13). Our results confirm that obstetric stressors such as PROM, prolonged labor, and repeated vaginal examinations increase the risk of intrauterine inflammation, while mechanical stress and tissue hypoxia may further amplify cytokine release and sterile inflammation (14, 15). Regarding immunological markers, this study directly compared IL-6, SII, and NLR. IL-6, a key acute-phase cytokine, was the most sensitive indicator of systemic inflammatory response but is limited by testing complexity and cost (12). Although WBC, CRP, and TNF-*α* were elevated in febrile women, they lacked independent predictive value after adjustment. Collectively, these findings underscore the prognostic importance of refined immunologic indices, with IL-6 offering sensitivity and SII providing greater specificity and practicality for maternal risk stratification (16, 17).

To date, predictive models for maternal fever remain scarce. Existing tools have primarily address complications such as postpartum hemorrhage, cesarean delivery, or preterm birth (18, 19). Our nomogram, ntegrating both labor-related clinical features and inflammation-based biomarkers, provides a novel approach for individualized fever risk prediction. Its graphical interface and reliance on readily available inputs further enhance its clinical utility. The findings suggest that intrapartum fever arises from a confluence of obstetric stress and inflammatory imbalance. PROM and repeated vaginal examinations increase the risk of ascending infection and immune activation. Prolonged labor may lead to hypoxia-induced cytokine expression. IL-6 directly mediates fever through hypothalamic activation, while SII and NLR reflect a systemic inflammatory shift characterized by neutrophil predominance and lymphocyte suppression—a profile commonly seen in both infectious and sterile inflammatory states. Our nomogram demonstrated excellent discrimination (AUC 0.910 in the training set and 0.906 in the validation set), exceeding the performance typically reported for single inflammatory markers and simple physiologic indices. For example, prior work that focused on a single hematologic biomarker such as the neutrophil-to-lymphocyte ratio (NLR) showed more modest discrimination in predicting epidural-related intrapartum fever, whereas our study further showed that systemic immune-inflammation index (SII) outperformed NLR as a single marker (AUC 0.846 vs. 0.716) and contributed substantially to the final model (13). Studies using non-laboratory physiologic indicators (e.g., pulse perfusion index) provide pragmatic tools but generally achieve lower accuracy compared with models integrating both obstetric and inflammatory variables (19). More recently, multicenter machine-learning approaches have reported strong performance; however, they often rely on complex feature engineering and may be less transparent for bedside use (18). In contrast, our nomogram balances accuracy, interpretability, and feasibility by combining readily available clinical features with robust inflammatory indices, and it shows strong internal validity with good calibration and decision-curve utility.

Intrapartum fever has been associated with several adverse maternal and neonatal outcomes, including increased risks of cesarean delivery, postpartum hemorrhage, neonatal encephalopathy, and neonatal sepsis evaluations (10, 11). These detrimental effects highlight the importance of early recognition and prevention. Management strategies primarily focus on supportive care and timely intervention, such as maternal hydration, antipyretic therapy, and optimizing labor management. In cases where intrauterine infection is suspected, prompt administration of broad-spectrum antibiotics is recommended to minimize maternal and neonatal morbidity (10). Against this background, our findings carry significant clinical implications. The predictive model developed herein allows for early identification of parturients at increased risk of developing intrapartum fever, enabling tailored monitoring and proactive management strategies. Limiting unnecessary vaginal examinations, optimizing labor progression, and considering early anti-inflammatory interventions in high-risk cases may reduce the incidence of maternal fever and its associated neonatal consequences. Furthermore, SII and NLR, due to their accessibility and low cost, may serve as useful biomarkers in real-world obstetric care. In clinical practice, this nomogram may be applied at the time of admission or during labor to stratify the risk of intrapartum fever. Women identified as high-risk could benefit from closer maternal and fetal monitoring, more judicious use of vaginal examinations, and timely administration of antipyretics or intravenous fluids. In cases with a strong suspicion of intrauterine infection, early initiation of prophylactic or therapeutic antibiotics may be warranted in line with current guidelines. Moreover, early recognition of high-risk patients may facilitate timely communication with neonatal teams and preparation for possible interventions such as NICU admission, ultimately improving perinatal outcomes.

Several limitations must be acknowledged. First, the retrospective design limits causal inference and is susceptible to residual confounding. Potential confounders such as prolonged rupture of membranes, maternal comorbidities (e.g., diabetes, hypertension, thyroid disorders), and obstetric interventions may not have been fully accounted for despite multivariate adjustment. Second, we were unable to differentiate between infectious and non-infectious etiologies of fever due to lack of microbiological and histological data. And inflammatory markers were only measured at admission, without dynamic assessment during labor, precluding evaluation of temporal trends. Third, the study was conducted in a single center, which may limit the generalizability of our findings to other obstetric populations and practice settings. The absence of external validation limits the robustness and clinical applicability of the nomogram beyond our cohort. Future studies should consider prospective designs, external validation, and real-time monitoring of cytokine profiles. In addition to IL-6, SII, and NLR, other biomarkers such as C-reactive protein (CRP), procalcitonin, IL-8, and placental or amniotic fluid cytokine profiling (e.g., TNF-*α*, chemokines) have been reported to evaluate systemic and intrauterine inflammatory responses during intrapartum fever (10, 15, 20). Although these markers were not included in the present study, their incorporation in future prospective research may further refine mechanistic understanding and improve predictive accuracy.

## Conclusion

Intrapartum fever is associated with both obstetric stress and systemic inflammation, as reflected by clinical and laboratory findings. We developed and validated a predictive nomogram incorporating readily accessible clinical and inflammatory markers, including IL-6, SII, and NLR. The model demonstrated strong discriminatory and calibration performance, enabling individualized risk assessment. This tool may assist clinicians in the early identification of high-risk parturients, thereby supporting timely clinical intervention and optimized intrapartum management.

## Data Availability

The authors acknowledge that the data presented in this study must be deposited and made publicly available in an acceptable repository, prior to publication. Frontiers cannot accept a manuscript that does not adhere to our open data policies.
